# Neural stem cells for disease modeling of Wolman disease and evaluation of therapeutics

**DOI:** 10.1186/s13023-017-0670-9

**Published:** 2017-06-28

**Authors:** Francis Aguisanda, Charles D. Yeh, Catherine Z. Chen, Rong Li, Jeanette Beers, Jizhong Zou, Natasha Thorne, Wei Zheng

**Affiliations:** 1 National Center for Advancing Translational Sciences, National Institutes of Health, Bethesda, MD USA; 2 iPSC Core, National Heart, Lung, and Blood Institute, National Institutes of Health, Bethesda, MD USA; 3 Present Address: Institute for Stem Cell Biology and Regenerative Medicine, Stanford University School of Medicine, Stanford, CA USA; 4 Present Address: Center for Devices and Radiological Health, Food and Drug Administration, Silver Spring, MD USA

**Keywords:** Wolman disease, Lysosomal storage disease, Induced pluripotent stem cells, Neural stem cells, Cell-based disease model

## Abstract

**Background:**

Wolman disease (WD) is a rare lysosomal storage disorder that is caused by mutations in the *LIPA* gene encoding lysosomal acid lipase (LAL). Deficiency in LAL function causes accumulation of cholesteryl esters and triglycerides in lysosomes. Fatality usually occurs within the first year of life. While an enzyme replacement therapy has recently become available, there is currently no small-molecule drug treatment for WD.

**Results:**

We have generated induced pluripotent stem cells (iPSCs) from two WD patient dermal fibroblast lines and subsequently differentiated them into neural stem cells (NSCs). The WD NSCs exhibited the hallmark disease phenotypes of neutral lipid accumulation, severely deficient LAL activity, and increased LysoTracker dye staining. Enzyme replacement treatment dramatically reduced the WD phenotype in these cells. In addition, δ-tocopherol (DT) and hydroxypropyl-beta-cyclodextrin (HPBCD) significantly reduced lysosomal size in WD NSCs, and an enhanced effect was observed in DT/HPBCD combination therapy.

**Conclusion:**

The results demonstrate that these WD NSCs are valid cell-based disease models with characteristic disease phenotypes that can be used to evaluate drug efficacy and screen compounds. DT and HPBCD both reduce LysoTracker dye staining in WD cells. The cells may be used to further dissect the pathology of WD, evaluate compound efficacy, and serve as a platform for high-throughput drug screening to identify new compounds for therapeutic development.

**Electronic supplementary material:**

The online version of this article (doi:10.1186/s13023-017-0670-9) contains supplementary material, which is available to authorized users.

## Background

Wolman disease is a rare lysosomal storage disorder with an incidence rate of less than 1 in 100,000 births [1]. WD is caused by mutations in the gene encoding lysosomal acid lipase (LAL), which results in nonfunctional levels of LAL activity. This leads to the accumulation of triglycerides (TG) and cholesteryl esters (CE) in the lysosomes of many cells and tissues [2]. Clinical manifestations include adrenal calcification, hepatosplenomegaly, and enlarged lymph nodes [3]. These organ and tissue enlargements are due to the accumulation of TG and CE, which may also occur in the intestine and central nervous system [4–6]. Typical life expectancy of patients without treatment is less than one year of age, with death due to multi-organ failure.

Complications of WD are thought to be related to malabsorption. Parenteral hyperalimentation has been shown to slow patient deterioration but not cure the disease [7]. Hematopoietic stem cell transplantation has been moderately successful for treating WD, but the procedure remains risky [8, 9]. Enzyme replacement therapy (ERT)—with sebelipase alfa (KANUMA®)—has recently been approved for treating WD [10, 11]. While ERT has reduced abdominal distention, hepatosplenomegaly, vomiting and diarrhea, and improved survival and weight gain, the long term effects of ERT for WD have yet to be evaluated [12]. Small molecule therapies have several advantages over recombinant enzymes—including lower production costs, more convenient administration, and the ability to penetrate the blood brain barrier. Therefore, the discovery and development of small molecule drugs might improve therapeutic efficacy and long term outcomes.

Recent advances in induced pluripotent stem cells (iPSCs) technology has enabled the generation of cell-based disease models derived from patient iPSCs. Several such cell-based models have been described for lysosomal storage diseases including Gaucher disease, Niemann Pick disease type A (NPA), Niemann Pick disease type C (NPC), and Pompe disease [13]. These models exhibit the disease phenotypes in cell culture systems and can be used to evaluate drug efficacy. In this study, we have generated four iPS cell lines from two WD patient fibroblasts. We subsequently generated neural stem cells (NSCs) from these WD iPSCs and found both lysosomal enlargement and neutral lipid accumulation in these WD NSCs. Using this cell-based WD model, we then evaluated the pharmacological effects of δ-tocopherol (DT) and hydroxypropyl-beta-cyclodextrin (HPBCD), which have been shown to reduce cholesterol storage in NPC cells [14, 15]. The results demonstrate that this cell-based WD model can be used for evaluating lead compounds and for compound screening in drug development.

## Methods

### Materials

CELLstart CTS substrate (#A1014201), GlutaMAX (#25030081), Nile red (#N-1142), LysoTracker Red DND-99 (#L-7528), Hoechst 33,342 trihydrochloride (#H3570), Neural Induction Medium (#A1647081), Essential 8 Medium (#A1517001), Human Neural Stem Cell Immunocytochemistry Kit (#A24354), StartingBlock BSA (#37579), TrypLE Express (#12605–036), Oct4 antibody (#A13998), AlexaFluor 488 Donkey anti-mouse antibody (#A21202), AlexaFluor 594 Donkey anti-rabbit antibody (#A21207), and AlexaFluor 595 Donkey anti-goat antibody (#A11058), Amplex red cholesterol assay kit (#A12216), LIPA monoclonal antibody clone 9G7F12,7G6D7 (#MA5–15278), low density lipoprotein from human plasma (LDL, # L3486) were purchased from ThermoFisher (Waltham, MA). Antibodies for SOX2 (#MAB2018), SSEA4 (#4755S), Nanog (#4903), and Tra-1-60 (#4746) were purchased from Cell Signaling (Danvers, MA). 96-well plates (#655090) were purchased from Greiner Bio-One (Monroe, NC). Fetal bovine serum (FBS, #SH30071.03) was purchased from GE Healthcare Life Sciences (Marlborough, MA). Matrigel (#354230) and six-well plates (#3506) were purchased from Corning (Tewksbury, MA). ROCK inhibitor Y-27632 (#1254) was purchased from Tocris Bioscience (Ellisville, MO). SOX1 antibody (# AF3369) was purchased from R&D Systems (Minneapolis, MN). Paraformaldehyde (#15714-S) was purchased from Electron Microscopy Services (Hatfield, PA). Monoclonal mouse-anti-LAMP2 antibody, clone H4B4, was purchased from Developmental Studies Hybridoma Bank (DSHB, Iowa City, IW). Dermal fibroblast lines were purchased from the Coriell Cell Repository (Camden, NJ), including one female WD patient line (GM11851) and one male WD patient line (GM06144).

### Generation and characterization of iPSCs

Primary skin fibroblasts from two patients with WD (GM11851 and GM06144) were obtained from the Coriell Cell Repository and reprogrammed by the NHLBI iPSC Core facility using the non-integrating CytoTune™-iPSC 2.0 Sendai viral vector kit (ThermoFisher) as previously described [16]. Briefly, fibroblasts were plated in one well of a 48-well plate and infected according to the kit instructions. After 4 days, cells were re-plated onto a Matrigel (Corning) coated 48-well plate in reprogramming medium, which is essentially Essential 8 Medium without TGF-beta. Cells were fed every other day until day 20. iPSCs were then picked and maintained in Matrigel-coated plates (Corning) with Essential 8 Medium (ThermoFisher). The healthy donor control iPSC line, NCRM-1, was previously generated by the NIH Center for Regenerative Medicine (CRM) from healthy donor CD34+ cord blood cells that were then reprogrammed using an episomal plasmid reprogramming method [17]. All iPSCs were passaged using an EDTA-based protocol as previously described [18]. Briefly, cells were washed twice and incubated with 0.5 mM EDTA in PBS buffer for 3–5 min. After aspirating EDTA/PBS, the cells were then disassociated by pipetting with Essential 8 Medium and reseeded onto a new Matrigel-coated plate containing Essential 8 Medium with 10 μM Y-27632 ROCK inhibitor (Tocris Bioscience). iPSC cultures were passaged beyond passage 15 to ensure complete clearance of Sendai virus and stability of the cell lines.

To confirm normal chromosomal integrity, WD iPSCs were analyzed by G-Banding karyotyping (WiCell Research Institute). Briefly, cells were incubated with colcemid and ethidium bromide, placed in a hypotonic solution, and then fixed. Cell preparations in metaphase were stained with Leishman’s stain. Twenty randomly selected metaphase cells were analyzed by G-banding for each line.

STR DNA analysis was performed by the WiCell Research Institute on each set of WD fibroblast cell lines, iPSC lines, and NSC lines to confirm their identity as derivatives of patient fibroblast lines GM11851 and GM06144.

To examine iPSC marker expression, cells were disassociated via the aforementioned EDTA-based passaging method and seeded into clear bottom, black, 96-well plates with Essential 8 Medium containing 10 μM of ROCK inhibitor. After incubating 24 h at 37 °C, media was removed and cells were fixed in 3.2% PFA for 30 min at room temperature. Cells were permeabilized with 0.3% Triton in PBS for 10 min at 4 °C. StartingBlock BSA (ThermoFisher) was added for 1 h. at room temperature, followed by an overnight incubation at 4 °C with the following primary antibodies diluted in blocking buffer: SOX2 (1:50 dilution, Cell Signaling), Oct4 (1:400 dilution, ThermoFisher), SSEA4 (1:1000 dilution, Cell Signaling), Nanog (1:400 dilution, Cell Signaling), and Tra-1-60 (1:500 dilution, Cell Signaling), with SOX1/SOX2 being co-stained in pairs. Cells were washed three times with DPBS and conjugated with the appropriate secondary antibodies diluted in blocking buffer for 1 h. at room temperature: AlexaFluor 595 Donkey Anti-Goat (ThermoFisher), AlexaFluor 488 Donkey Anti-Mouse (ThermoFisher), AlexaFluor 594 Donkey Anti-Rabbit (ThermoFisher). Plates were imaged using an IN Cell Analyzer 2200 (GE Healthcare).

### NSC differentiation and characterization

To initiate differentiation, 2.5 × 10^5^ iPSCs were seeded into one Matrigel-coated well in a six-well plate with 2.0 mL Essential 8 medium and 10 μM ROCK inhibitor. After 24 h, the medium was switched to 2.5 mL of Pluripotent Stem Cell Neural Induction Medium—Neurobasal medium with 1X Neural Induction Supplement (ThermoFisher). Induction medium was changed every day for 7 days. On the seventh day, NSCs were disassociated using TrypLE Express (ThermoFisher), passed through a 40 μm cell strainer, and then centrifuged for 4 min at 300 × g. NSCs were reseeded in Neural Expansion Medium—equal parts Advanced DMEM/F12 and Neurobasal medium with 1X Neural Induction Supplement—with 10 μM ROCK inhibitor in a T25 flask.

At the third passage, NSCs were seeded into a 96-well plate with 10 μM ROCK inhibitor for immunocytochemistry characterization using the Human Neural Stem Cell Immunocytochemistry Kit (ThermoFisher). After 24 h at 37 °C, media was removed and cells were fixed in 4% PFA in DPBS for 15 min at room temperature. Cells were permeabilized with 0.5% Triton X-100 in DPBS for 15 min at room temperature. Blocking buffer (3% BSA in DPBS) was added for 1 h at room temperature, followed by an overnight incubation at 4 °C with the following primary antibodies diluted in blocking buffer: Nestin (1:50 dilution), PAX6 (1:50 dilution), SOX1 (1:50 dilution), and SOX2 (1:50 dilution), with Nestin/SOX2 and SOX1/PAX6 being co-stained in pairs. Cells were washed and incubated with the appropriate secondary antibodies from the Human Neural Stem Cell Immunocytochemistry Kit diluted in blocking buffer for 1 h. at room temperature: AlexaFluor 595 donkey anti-goat (1:250), AlexaFluor 488 donkey anti-mouse (1:250), AlexaFluor 594 donkey anti-rabbit (1:250). Cells were washed and incubated for 10 min at room temperature with NucBlue diluted in DPBS. Plates were imaged using an IN Cell Analyzer 2200 using the appropriate filter sets.

### Western blot analysis

NSCs were seeded on 10-cm dishes with 10 μM ROCK inhibitor-supplemented media. The next day media was changed to remove ROCK inhibitor. Media was changed again the following day, and the cells were grown for another 48 h. Cells were harvested using M-PER™ Mammalian Protein Extraction Reagent (ThermoFisher) containing protease inhibitors (Roche) with scraping. Cell debris was pelleted at 13,200 × g for 5 min at 4 °C, and protein content was quantitated in the lysates. 15 μg of lysate from each cell line was resolved on a 4–12% NuPAGE Novex gel (ThermoFisher), and transferred using the iBlot Dry Blotting System (ThermoFisher). Blots were blocked with Starting Block buffer (ThermoFisher), incubated with primary antibody mouse-anti-LIPA clone 9G7F12,7G6D7 (ThermoFisher), and with secondary antibody goat-anti-mouse-HRP (Cell Signaling Technology, CST), then developed. Blots were then stripped and GAPDH expression was evaluated as a loading control with rabbit-anti-GAPDH (CST) primary antibody and goat-anti-rabbit-HRP (CST) secondary antibody.

### LAL activity assay

NSCs were seeded onto 10-cm dishes and grown as described for immunoblot analysis. On the day of harvest, cells were washed with DPBS and harvested in lysis buffer (0.5% Triton X-100 with protease inhibitor cocktail, Roche) with scraping. Cell lysates were pelleted at 13,200 × g for 5 min at 4 °C. The supernatant was removed and frozen at −80 °C until assayed. The LAL activity assay was modified from Guy et al. [19]. Briefly, 4-methylumbelliferyl palmitate (4-MUP, Cayman Chemicals) was dissolved to 400 μM in 1.45% (*v*/v) Triton X-100 with heating at 68 °C. Cardiolipin sodium salt (Sigma-Aldrich) was dissolved in ethanol to 5 mg/mL. The substrate solution consisted of 133 μM 4-MUP, 0.65% Triton X-100 and 0.33 mg/mL cardiolipin in 67 mM acetate buffer pH 4.0. Reactions were initiated with the combination of 10 μL lysate (1 μg protein) and 30 μL of substrate solution. After 20 min at 37 °C, the reaction was terminated by adding 60 μL 5% perchloric acid, then 200 μL of 1 M sodium bicarbonate-sodium carbonate buffer pH 9.5. Fluorescence signal, with Ex/Em of 320/460 nm, was measured on an EnVision Multilabel Reader (PerkinElmer, Waltham, MA).

### Nile red and LysoTracker staining

10,000 cells/well NSCs were seeded in black, clear bottom, CELLstart coated 96-well plates with 10 μM ROCK inhibitor and grown overnight. The following day, the media was removed and the cells were then treated with 10% FBS-supplemented media for another 48 h. Cells were then stained with either Nile red or LysoTracker dye.

For Nile red staining, NSCs were incubated at 37 °C for 10 min with 1 μM Nile red (ThermoFisher) in media without FBS to visualize neutral lipid storage. Cells were washed three times with DPBS containing calcium and magnesium and then fixed at room temperature for 25 min with 3.2% paraformaldehyde solution (Electron Microscopy Services) containing 0.3 μg/mL Hoechst 33,342 (ThermoFisher) diluted in DPBS. Cells were washed once with DPBS and then imaged with an IN Cell Analyzer 2200.

For LysoTracker staining, NSCs were incubated at 37 °C for 1 h with 50 nM LysoTracker Red (ThermoFisher) in media without FBS to visualize lysosomes. Cells were washed three times with DPBS with calcium and magnesium and then fixed at room temperature for 25 min with 3.2% paraformaldehyde solution containing 0.3 μg/mL Hoechst 33,342 diluted in DPBS. Cells were washed once with DPBS and then imaged with an IN Cell Analyzer 2200.

### LAMP2 Immunocytochemistry

NSCs were seeded in 96-well plates and incubated with 10% FBS as described for the LysoTracker and Nile red assays. For LAMP2 immuno-staining, cells were washed with DPBS with calcium and magnesium, and then fixed with 3.2% paraformaldehyde for 20 min at room temperature. Wells were washed, permeabilized with 0.5% Triton X-100 for 15 min, then washed and incubated for 1 h with Cell Staining Buffer (BioLegend), before incubating overnight with primary anti-LAMP2 antibody at 1:200 dilution (Developmental Studies Hybridoma Bank) at 4 °C. Cells were then washed with DPBS and incubated with AlexaFluor 488 anti-mouse antibody (ThermoFisher) at 1:250 dilution for 1 h at room temperature. Secondary antibody solution was washed away and cells were stained with Hoechst solution in DPBS and then imaged with an IN Cell Analyzer 2200.

### Image and data analysis

Image sets for each experiment were analyzed using the IN Cell Investigator Analysis Modules. Protocols were developed to identify nuclei, lysosomes, and neutral lipids. Data was reported as the average integrated intensity per cell per well, calculated as the product of object pixel intensity and area. Data was graphed with standard deviation (SD), and significance was calculated using the unpaired, double-tailed Student’s t-test. Significance represented as: * as *p* ≤ 0.05, ** as *p* ≤ 0.01, *** as *p* ≤ 0.001, and **** as *p* ≤ 0.0001. All image analysis was performed using 3 replicate wells with 9 image fields/well.

### LDL treatment

NSCs were seeded in 96-well plates and grown overnight as described for the LysoTracker and Nile red assays. The following day, the media was removed and the cells were treated with 15 μg/mL LDL (ThermoFisher) in media for another 48 h. After incubating 48 h with LDL, the cells were prepared for LysoTracker or Nile red assays as described.

### Amplex red esterified cholesterol assay

Cells were seeded in 96-well plates and incubated with 10% FBS as described for the LysoTracker and Nile red assays, then assayed for CE levels using the Amplex red cholesterol assay kit (ThermoFisher). Briefly, cells were washed once with HBSS and the buffer was removed. To each well, 50 μL PBS plus 50 μL of working solution was added. The working solution was prepared according to manufacturer’s directions and contained 0.3 mM Amplex Red, 2 U/mL cholesterol oxidase, 2 U/mL horseradish peroxidase, 0.1 M potassium phosphate pH 7.4, 0.05 M sodium chloride, 5 mM cholic acid, 1% Triton X-100, with and without 0.2 U/mL cholesterol esterase. Microplates were centrifuged at 200 × g for 3 min, and then incubated for 1 h at 37 °C. Fluorescence signal was measured using a Tecan Infinite M1000 monochromator with excitation/emission of 560/590 nm, with 10 nm bandwidths. Reported relative fluorescence units (RFU) represent the fluorescence values of samples with cholesterol esterase treatment minus values without cholesterol esterase treatment. Two replicate wells were averaged for each condition.

### Recombinant human LAL treatment

Recombinant human LAL (rhLAL) enzyme was custom purchased from MyBioSource (San Diego, CA). Briefly, amino acids 22–399 of human LAL were cloned, his-tagged, expressed in mammalian cells, and purified over a Ni-NTA column. For the rhLAL assay, NSCs were seeded at 10,000 cells/well in black, clear bottom, 96-well plates with CELLstart coating and 10 μM ROCK inhibitor. After 4 h incubation at 37 °C, the media was removed and cells were treated with rhLAL diluted in media without ROCK inhibitor. Cells were incubated overnight at 37 °C and then washed three times with media to remove excess enzyme. The cells were then treated with 10% FBS media for 48 h at 37 °C, after which Nile red or LysoTracker staining was performed.

### Compound treatment

Cells were seeded at 10,000 cells/well in black, clear bottom, 96-well plates with CELLstart coating and 10 μM ROCK inhibitor and then grown overnight. Media was then replaced with media without ROCK inhibitor and cells were pretreated with compound for 24 h. The medium was then replaced with a medium containing 10% FBS and the same compound. After an additional 48 h incubation, NSCs were stained with either Nile red or LysoTracker dye.

### ATP content cytotoxicity assay

Cells were seeded in 96-well plates and treated with compounds as in compound treatment experiments. After the 48 h incubation with compounds, ATPLite 1step Luminescence assay reagent (PerkinElmer) was added according to manufacturer’s directions. The one step lysis and detection reagent generates luminescence signal, which was read on a ViewLux plate reader (PerkinElmer). Data was normalized as vehicle treated NSC wells as 100% viability, and media only wells as 0% viability. Data presented is the average of 2 replicate wells.

## Results

### Generation of iPSCs from WD patient fibroblasts and subsequent NSC differentiation

**Fig. 1:**
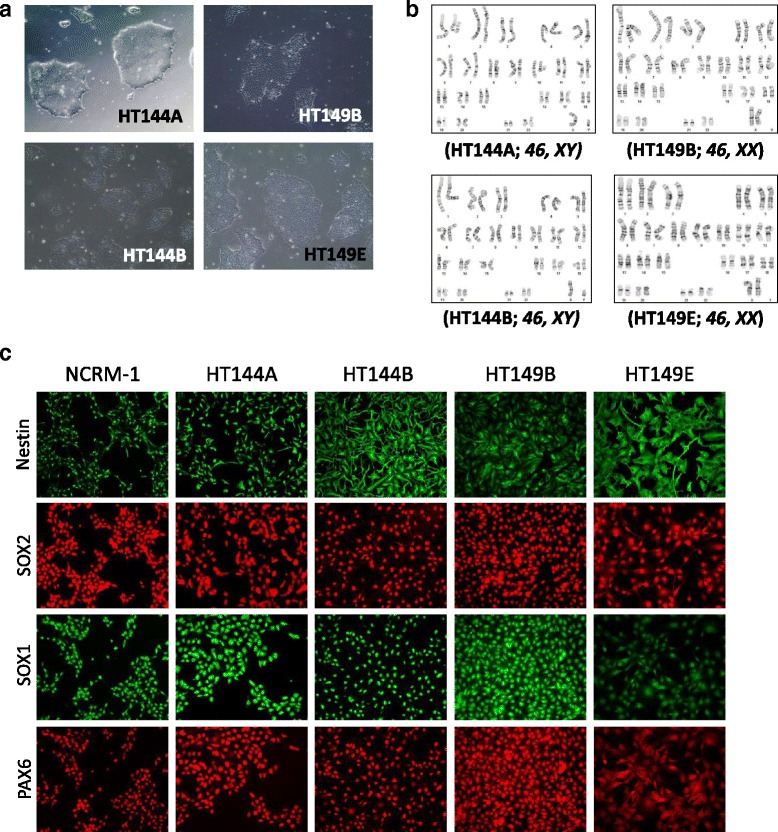
Characterization of WD patient-derived iPSCs and NSCs. **a** Images of WD iPSC colonies cultured on Matrigel. **b** iPSCs derived from WD patient fibroblasts display normal karyotypes. **c** Characterization of NSCs derived from WD and healthy donor control iPSCs, showing expression NSC markers Nestin, SOX1, SOX2, and PAX6

Two WD patient fibroblast lines, GM06144 and GM11851, were used to generate four iPSC clones, with GM06144 giving rise to HT144A and HT144B, and GM11851 giving rise to HT149B and HT149E (Fig. 1a). Immunocytochemistry staining showed that the four WD iPSC lines expressed the pluripotency markers SOX2, Nanog, Oct4, Tra-1-60, and SSEA4 (Additional file 1: Figure S1). WD iPSC lines showed normal karyotype (Fig. 1b), and STR DNA analysis confirmed that their identities matched their parental fibroblasts (Additional file 1: Figure S2). In addition to the four WD iPSC lines generated, a previously characterized iPSC line derived from a healthy donor, NCRM-1, was used as a control. The WD and control iPSCs were then differentiated to NSCs and confirmed to express the essential NSC markers Nestin, SOX1, SOX2, and PAX6 (Fig. 1c). In general, the efficiency of NSC generation from iPSCs for all five lines was on par with other NSCs our lab has generated. While we did not observe any abnormal morphology or proliferation rates in three of the WD NSCs, the HT149E NSCs grew significantly slower compared with other WD and control NSCs. For these reasons, we did not carry out further experiments with the HT149E NSC line.

### WD NSCs show reduced LAL activity

**Fig. 2:**
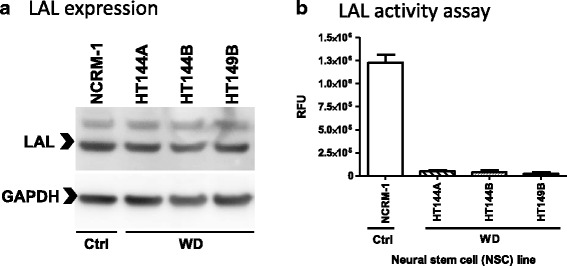
LAL expression and activity levels in WD NSCs. **a** Western blot images of LAL and GAPDH loading control expression levels in WD and control NSCs. **b** LAL activity assay using 4-MUP substrate. Activity presented in relative fluorescence unit (RFU)

Wolman disease patient cells have been reported to show reduced LAL activity. To evaluate whether this hallmark feature of the disease is maintained in the patient-derived NSCs, LAL expression and activity were examined in the WD NSCs and compared to control NSCs. Western blot analysis of LAL protein levels in NSCs showed similar protein expression between the healthy donor controls and the WD NSCs (Fig. 2a). However, all three WD NSCs showed dramatically decreased levels of LAL activity that represent over 20-fold reduction from control NSCs (Fig. 2b). These results are consistent with previous reports that while LAL expression remains unchanged, LAL activity is reduced in WD fibroblasts [2, 20].

### WD NSCs exhibit increased LysoTracker dye staining and lipid accumulation

**Fig. 3:**
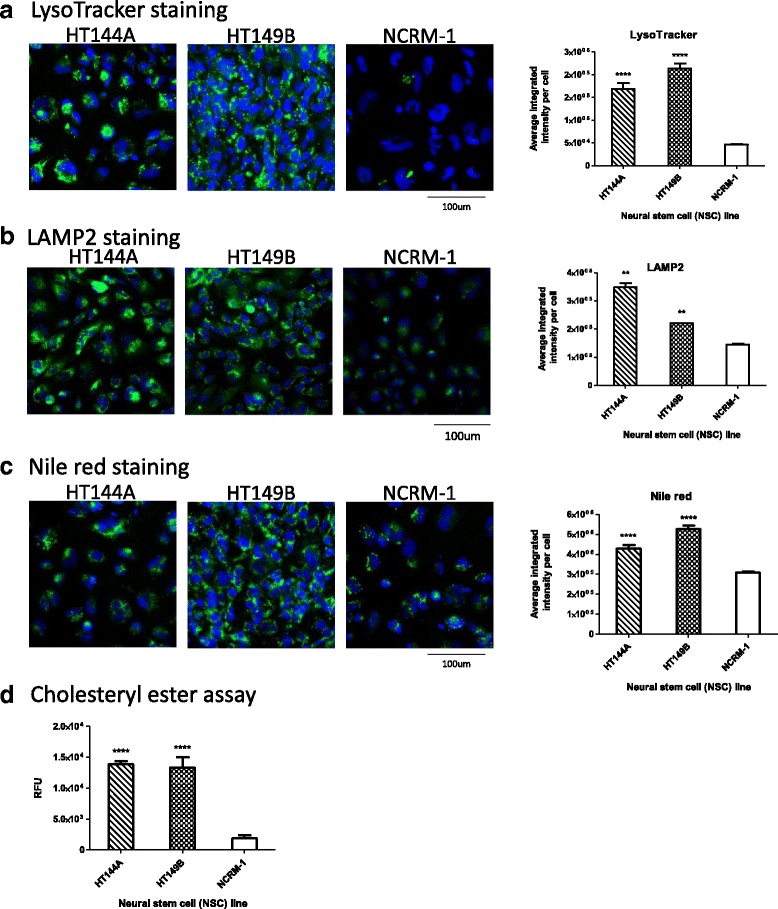
WD NSCs show increased lysosomal staining and lipid accumulation. **a** LysoTracker (*green*) and nuclear (*blue*) staining images with quantitation of WD and control NSCs. **b** LAMP2 (*green*) and nuclear (*blue*) staining images with quantitation of WD and control NSCs. **c** Nile red (*green*) and nuclear (*blue*) staining images with quantitation of WD and control NSCs. **d** Amplex red assay for cholesteryl ester content of WD and control NSCs. All data are displayed as mean ± SD. Statistical significance was calculated as each WD cell line compared against NCRM-1

A hallmark of WD is the accumulation of CE and TG in the lysosomes of many tissues [21]. To evaluate the storage of these substrates in the WD patient cells, the Nile red dye was used to stain accumulated neutral lipids and the Amplex red cholesterol assay was used to assess the CE accumulation in these cells [22]. In addition, LysoTracker dye and LAMP2 immunocytochemistry were used to visualize lysosomal changes. It has been previously reported that WD fibroblasts exhibit a strong yellow-gold Nile red staining, indicating accumulation of neutral lipids such as CE and TG, and have increased LysoTracker staining, indicating increased lysosomal content [23]. We examined the WD NSCs for similar phenotypes after the WD and control NSCs were cultured for 48 h in 10% FBS supplemented media to induce accumulation of CE and other lipids. All patient NSC lines showed increased LysoTracker staining, with HT144A NSCs showing 3.7-fold, HT144B NSCs 4.3-fold, and HT149B NSCs 4.6-fold increase compared to control NSCs (Fig. 3a, Additional file 1: Figure S4). While the patient NSCs HT144A and HT149B showed significant elevations in LAMP2 and Nile red staining compared with control NSCs, the levels were more moderate than that of LysoTracker staining. HT144A and HT149B showed a 2.4- and 1.5-fold increase, respectively, in LAMP2 staining (Fig. 3b), and a 1.4- and 1.7-fold increase, respectively, in Nile red staining (Fig. 3c). WD NSCs also showed increased signal in the Amplex red assay for CE content, with HT144A and HT149B showing 7.3- and 7.0-fold increase over NCRM-1 NSCs (Fig. 3d).

In addition to lipid loading using FBS, loading with LDL was also tested. LDL from human plasma was used at a concentration previously known to cause lipid storage and lysosomal enlargement in cell culture models of other lysosomal storage disorders [24].

Compared with FBS lipid loading, feeding with LDL resulted in lower fold increase between WD NSCs over NCRM-1 NSCs in LysoTracker staining, but higher fold increase in Nile red staining (Additional file 1: Figure S3).

### rhLAL reduces lysosomal size and lipid accumulation in WD NSCs

**Fig. 4:**
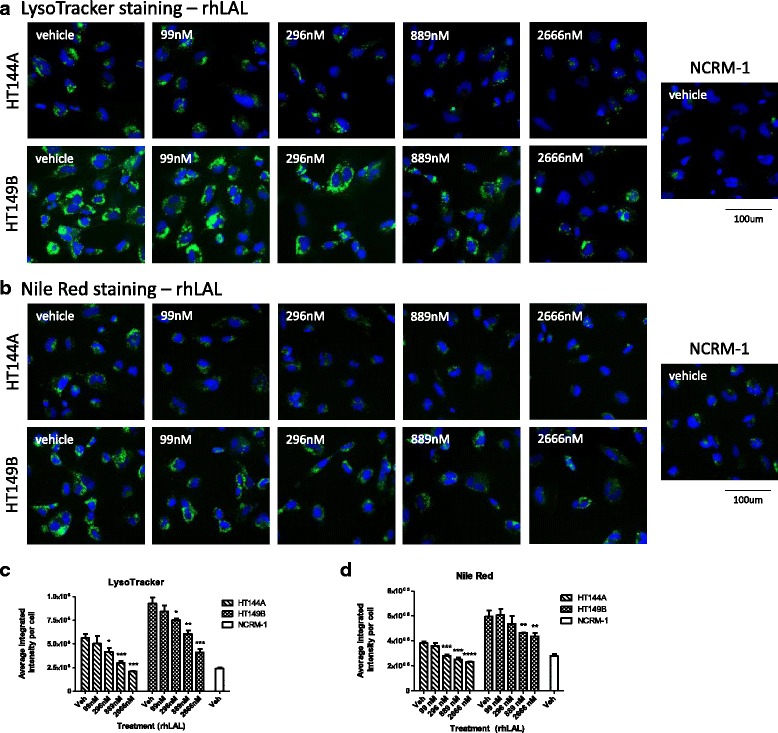
rhLAL reduces lysosomal staining and lipid accumulation in WD NSCs. **a** LysoTracker (*green*) and nuclear (*blue*) staining of rhLAL treated NSCs. **b** Nile red (*green*) and nuclear (*blue*) staining of rhLAL treated NSCs. **c** Quantitation of LysoTracker staining. **d** Quantitation of Nile red staining. Data are displayed as mean ± SD. Statistical significance was determined by comparing each rhLAL concentration treatment to vehicle treatment in the same WD cell line

The disease phenotypes in WD are caused by a severe deficiency in LAL [2]. To test whether these phenotypes can be reversed by ERT, we treated the WD NSCs with recombinant human LAL enzyme (rhLAL). The WD NSCs were pretreated with rhLAL and then challenged with 10% FBS media. Treatment with rhLAL significantly decreased lipid accumulation in a concentration dependent manner (Fig. 4). At the highest concentration tested of 2.67 μM rhLAL, LysoTracker staining was reduced by 63% in the HT144A NSCs and 56% in the HT149B NSCs (Fig. 4a, c). Similarly, Nile red staining decreased by 40% in the HT144A NSCs and 27% in the HT149B NSCs when treated with 2.67 μM rhLAL (Fig. 4b, d). The results indicated that rhLAL treatment effectively reduced lipid accumulation and reduced lysosomal staining in WD NSCs. No toxicity was observed at the highest concentration of 2.67 μM rhLAL treatment.

### Delta-tocopherol and HPBCD reduced lysosomal staining and CE accumulation in WD NSCs

**Fig. 5:**
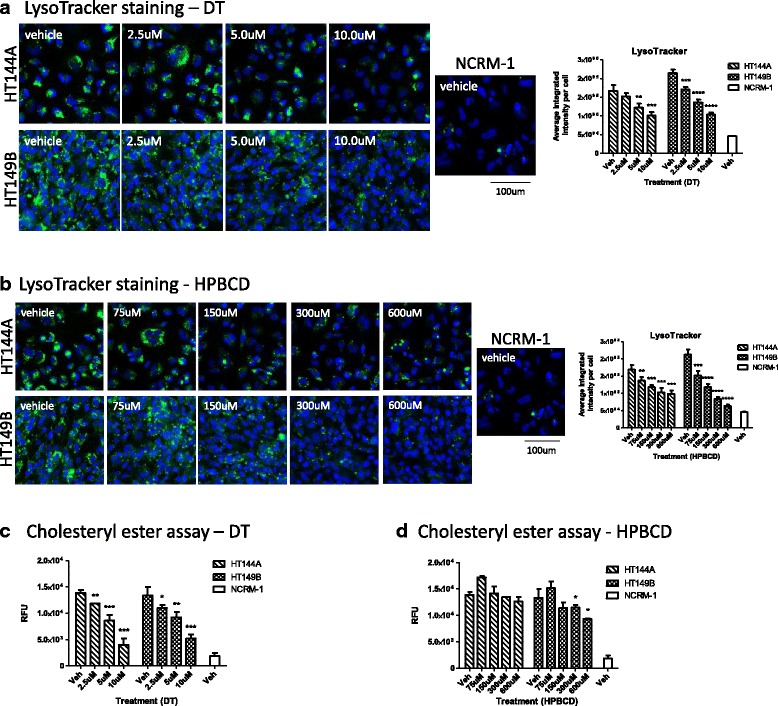
DT and HPBCD treatment reduces lysosomal staining in WD NSCs. **a** LysoTracker (*green*) and nuclear (*blue*) staining images with quantitation of DT-treated NSCs. **b** LysoTracker (*green*) and nuclear (*blue*) staining images with quantitation of HPBCD-treated NSCs. **c** Amplex red assay for cholesteryl ester content of WD NSCs treated with DT. **d** Amplex red assay for cholesteryl ester content of WD NSCs treated with HPBCD. Data are displayed as mean ± SD. Statistical significance was calculated as each concentration of compound compared against vehicle in the same WD cell line

DT and HPBCD are two structurally unrelated compounds (Additional file 1: Figure S5) that have been reported to reduce lipid accumulation in several fibroblast lines from patients of lysosomal storage diseases including WD [15, 25]. Thus, we tested DT’s effect on reducing lysosomal and lipid accumulations in the WD NSCs. We found that DT strongly reduced lysosomal staining in all three WD NSC lines in a dose-dependent manner (Fig. 5a, Additional file 1: Figure S6A). Compared to vehicle controls, treatment with 10 μM of DT resulted in a 39% reduction in LysoTracker staining in HT144A NSCs, 47% reduction in HT144B NSCs, and 52% reduction in HT149B NSCs. DT also strongly reduced CE accumulation in WD cells in a dose-dependent manner (Fig. 5c, Additional file 1: Figure S6B). Compared to vehicle controls, treatment with 10 μM of DT resulted in a 71% reduction in Amplex Red CE assay in HT144A NSCs, 67% reduction in HT144B NSCs, and 61% reduction in HT149B NSCs.

To evaluate the effects of HPBCD on ameliorating disease phenotypes in WD, we tested this compound in WD NSCs at concentrations ranging from 75 to 600 μM. We found that HPBCD reduced LysoTracker staining in all three WD NSC lines in a dose-dependent manner (Fig. 5b, Additional file 1: Figure S6C). When compared to vehicle controls, 600 μM HPBCD reduced LysoTracker staining by 42% in the HT144A NSCs, 22% in HT144B NSCs, and 70% in the HT149B NSCs. HPBCD also reduced CE accumulation in two of the WD cells, by 36% in HT144B and by 30% in HT149B (Fig. 5d, Additional file 1: Figure S6D).

DT and HPBCD showed weaker efficacy in reducing Nile red staining (neutral lipid accumulation) compared to their effect on reducing lysosomal staining (Additional file 1: Figure S7). The results show that DT did not reduce Nile red staining in either HT144A or HT149B cell lines (Additional file 1: Figure S7A). HPBCD showed variable effects of reducing Nile red staining by 34% at the highest concentration tested of 600 μM in HT149B NSCs, but showed no reduction of Nile red staining in HT144A cells (Additional file 1: Figure S7B). These data suggest that unlike ERT, DT and HPBCD are only partially able to correct the lysosomal and CE accumulation phenotypes, but do not normalize the accumulation of neutral lipids. It is possible that DT and HPBCD at higher concentrations might be able to fully reverse WD phenotypes, however, using such high concentrations resulted in cytotoxicity. When the cytotoxicity of DT and HPBCD was evaluated both visually and via an ATP content assay, loss of viability was seen at concentrations above 10 μM DT and 1200 μM HPBCD (Additional file 1: Figure S8A, B).

### WD disease phenotype reduced significantly by combination of delta-tocopherol and HBPCD in WD NSCs

**Fig. 6:**
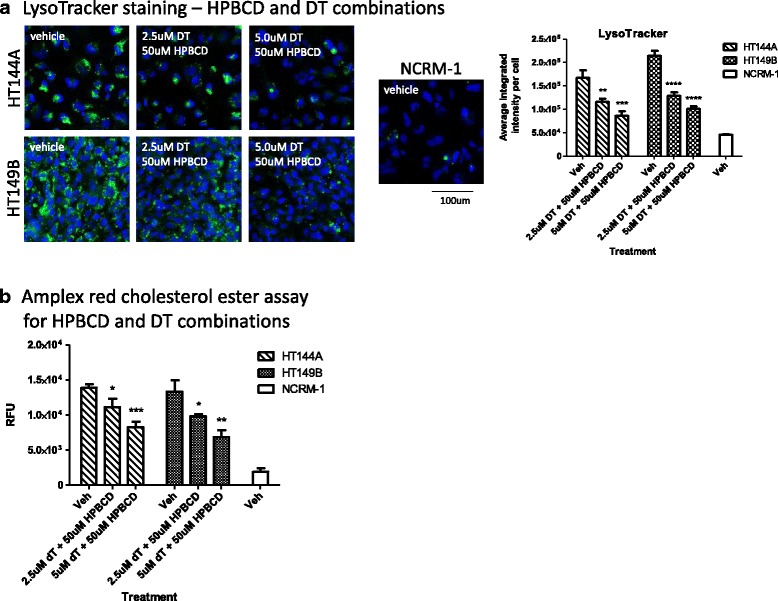
DT and HPBCD combination treatment have an additive effect on reducing lysosomal staining in WD NSCs. **a** LysoTracker (*green*) and nuclear (*blue*) staining images with quantitation of DT/HPBCD combination treatment. **b** Amplex red assay for cholesteryl ester content of WD NSCs treated with DT and HPBCD combination. Data are displayed as mean ± SD. Statistical significance was calculated as each combination treatment compared against vehicle treatment in the same WD cell line

The combination therapy of DT and HPBCD has previously been shown to have an additive effect on reducing cholesterol accumulation in NPC patient cells [25]. We tested this combination on WD NSCs. After treating WD NSCs with 5 μM DT and 50 μM HPBCD, LysoTracker staining was reduced by 48% in HT144A NSCs, 27% in HT144B NSCs, and 53% in HT149B NSCs (Fig. 6a, Additional file 1: S9A). The therapeutic effect of the combination therapy was improved compared with either of these compounds used alone in HT144A and HT149B, where treatment with 5 μM DT only reduced LysoTracker staining by 27% in WD patient line HT144A, and 36% in HT149B NSC (Fig. 5a), and the effect of 50 μM HPBCD treatment was minimal. These results indicate that DT has an additive effect with HPBCD in reducing LysoTracker staining in all WD NSCs. The combination of DT and HPBCD at these concentrations did not impact cell viability (Additional file 1: Figure S8C). However, similar to treatment with single agents, combination treatment of DT and HPBCD did not significantly alter the Nile red staining in any of the WD NSC lines (Additional file 1: Figure S7C). Combination treatment of DT and HPBCD also reduced CE accumulations in all three cell lines (Fig. 6b, Additional file 1: Figure S9B).

## Discussion

WD is a rare lysosomal storage disorder lacking effective small molecule therapies. KANUMA® is an ERT that was recently approved for the treatment of WD by the FDA [11]. However, ERTs generally have shortcomings such as inconvenient intravenous administration, inability to cross the blood-brain barrier, and high cost. It is possible that additional small molecule drugs might be developed to complement ERT by either increasing efficacy, and/or reducing the frequency of administration of ERT. In order to facilitate drug development for WD, we have generated four iPSC lines from two WD patient dermal fibroblast lines. These iPSCs were differentiated into NSCs that were shown to maintain key features of WD disease, such as loss of LAL activity (Fig. 2), accumulation of neutral lipids, and increased lysosomal staining (Fig. 3). The neutral lipid accumulation and increased lysosomal staining phenotypes were ameliorated by ERT with rhLAL in a dose-dependent manner (Fig. 4). While some recombinant enzymes have been shown to enter cells through the glucose-6-phosphate receptor [26], the exact mechanism for cell entry utilized by rhLAL remains unclear.

As a proof of concept, we tested DT and HPBCD in the WD NSC cell-based models. These two compounds have been found to be efficacious in other cell-based models of lysosomal storage disorders [15, 25]. Treatment with DT and HPBCD reduced lysosomal staining and CE content in the WD NSCs. These results demonstrated that patient iPSC derived WD NSCs can be used as a cell-based disease model for evaluating therapeutic effects of small molecules. The successful generation of WD iPSCs enables development of high-throughput screening assays in a patient derived cell-based model for drug discovery.

The result that rhLAL was able to correct both the lipid accumulation and increased lysosomal staining phenotypes, as indicated by Nile red and LysoTracker stains, while HPBCD and DT were only able to correct the lysosomal staining phenotype may suggest that these compounds are not able to reduce all forms of accumulated lipids in WD cells. This may be due to the differences in mechanism of action between rhLAL, DT, and HPBCD. While the exact mechanism of action of DT and HPBCD remains to be elucidated, initial studies indicate that these compounds increase intracellular calcium release and lysosomal exocytosis [15, 27]. Both of these compounds seem to function by increasing compensatory pathways to reduce both primary and secondary lipid accumulation in lysosomes. This is downstream of the effect exerted by rhLAL, which is to reduce and prevent the primary accumulation of CE and TG lipids. It is also possible that DT and HPBCD might be more efficacious at higher concentrations, but due to compound cytotoxicity observed in our experiments, this was not feasible to determine for both compounds. Additional screening with large compound collections may identify better lead compounds with similar efficacy to rhLAL.

While most clinical symptoms of WD are not related to the central nervous system, biopsies have shown lipid accumulation in the brain of WD patients [28, 29]. Impaired brain myelination was also reported, which may be caused by the abnormal CE and TG metabolism in WD [30]. We chose NSCs as a proof of concept cell-based model because the current technology for NSC differentiation from iPSC is more advanced and results in higher efficiency than that of other cell types. In addition, the successful development of WD patient-derived iPSCs opens the possibilities of future differentiation into other cell types and development of cell-based models that are more disease relevant.

The use of patient derived iPSCs to model disease phenotypes in vitro is a new approach for drug discovery and development. While WD animal models have previously been generated and found to recapitulate certain aspects of WD, these models do not provide the high degree of throughput necessary for drug screening as do the cell-based models [31]. The additional advantage of patient-derived iPSCs and their derivative cells over animal models is that the iPSC-derived cells have the same genetic mutations as in patients. However, a disadvantage is that cell-based models may not replicate the long term accumulation effects observed in patients or animal models due to their limited incubation time in cell culture. Therefore, the WD iPSCs and their derivative cells provide an effective phenotypic disease model for screening compounds for drug development. The final lead compounds should be evaluated in the animal model of Wolman disease before advancing to clinical trials in patients.

## Conclusions

Three lines of iPSCs have been generated from WD patient fibroblasts, and the NSCs derived from these WD iPSCs exhibit the hallmark disease phenotypes of lipid accumulation, severely deficient LAL activity, and increased LysoTracker dye staining. The rhLAL ERT effectively corrected disease phenotype in the WD NSCs. DT and HPBCD partially corrected the disease phenotype in WD NSCs with reduction of LysoTracker dye staining and CE accumulation. This cell-based WD model can be used for compound screening to identify lead compounds for small molecule drug development.

## Additional file

Additional file 1: Supplemental figures.
**Figure S1.** Immunocytochemical characterization of WD iPSCs. **Figure S2.** STR DNA analysis of WD fibroblasts, iPSCs, and NSCs. **Figure S3.** LysoTracker staining and Nile red staining of LDL loaded NSCs. **Figure S4.** HT144B NSCs show increased LysoTracker staining. **Figure S5.** Chemical structures. **Figure S6.** DT and HPBCD treatment reduces lysosomal staining in HT144B NSCs. **Figure S7.** DT and HPBCD do not significantly reduce neutral lipid accumulation in WD NSCs. **Figure S8.** High concentrations of DT and HPBCD affect cell viability. **Figure S9.** DT and HPBCD combination treatment have an additive effect on reducing lysosomal staining in HT144B NSCs. (PDF 652 kb)

## Abbreviations

CE: Cholesteryl esters
DT: Delta-tocopherol
ERT: Enzyme replacement therapy
HPBCD: Hydroxypropyl-beta-cyclodextrin
iPSCs: Induced pluripotent stem cells
NPC: Niemann Pick disease type C
NSCs: Neural stem cells
rhLAL: Recombinant human lysosomal acid lipase
TG: Triglycerides
WD: Wolman disease
