# Supplementary material for: Neural stem cells for disease modeling of Wolman disease and evaluation of therapeutics
Source: Orphanet J Rare Dis. 2017 Jun 28;12:120. doi: 10.1186/s13023-017-0670-9 (PMC5490176; doi:10.1186/s13023-017-0670-9)
Supplement: Additional file 1: Supplemental figures. — Figure S1. Immunocytochemical characterization of WD iPSCs. Figure S2. STR DNA analysis of WD fibroblasts, iPSCs, and NSCs. Figure S3. LysoTracker staining and Nile red staining of LDL loaded NSCs. Figure S4. HT144B NSCs show increased LysoTracker staining. Figure S5. Chemical structures. Figure S6. DT and HPBCD treatment reduces lysosomal staining in HT144B NSCs. Figure S7. DT and HPBCD do not significantly reduce neutral lipid accumulation in WD NSCs. Figure S8. High concentrations of DT and HPBCD affect cell viability. Figure S9. DT and HPBCD combination treatment have an additive effect on reducing lysosomal staining in HT144B NSCs. (PDF 652 kb) [file 13023_2017_670_MOESM1_ESM.pdf]

Supplemental material

Neural stem cells for disease modeling of Wolman disease and evaluation of therapeutics

Francis Aguisanda[1‡*], Charles D. Yeh[1*], Catherine Z. Chen[1], Rong Li[1], Jeanette Beers[2], Jizhong Zou[2], Natasha Thorne[1#†], and Wei Zheng[1†]

Author details

[1]National Center for Advancing Translational Sciences, National Institutes of Health, USA.

[2]Center for Molecular Medicine, National Heart, Lung, and Blood Institute, National Institutes of Health, USA.

‡Current affiliation: Institute for Stem Cell Biology and Regenerative Medicine, Stanford University School of Medicine, USA

[#]Current affiliation: Center for Devices and Radiological Health, Food and Drug Administration, USA.

*These authors contributed equally to this work

[†]Corresponding authors:

Wei Zheng, Ph.D., National Center for Advancing Translational Sciences, NIH, 9800 Medical Center Drive, Room 311, MSC 3375, Bethesda, MD 20892, USA.

Natasha Thorne, Ph.D., Center for Devices and Radiological Health, FDA, 10903 New Hampshire Avenue, Building 66, Room 5551, Silver Spring, Maryland 20993, USA.

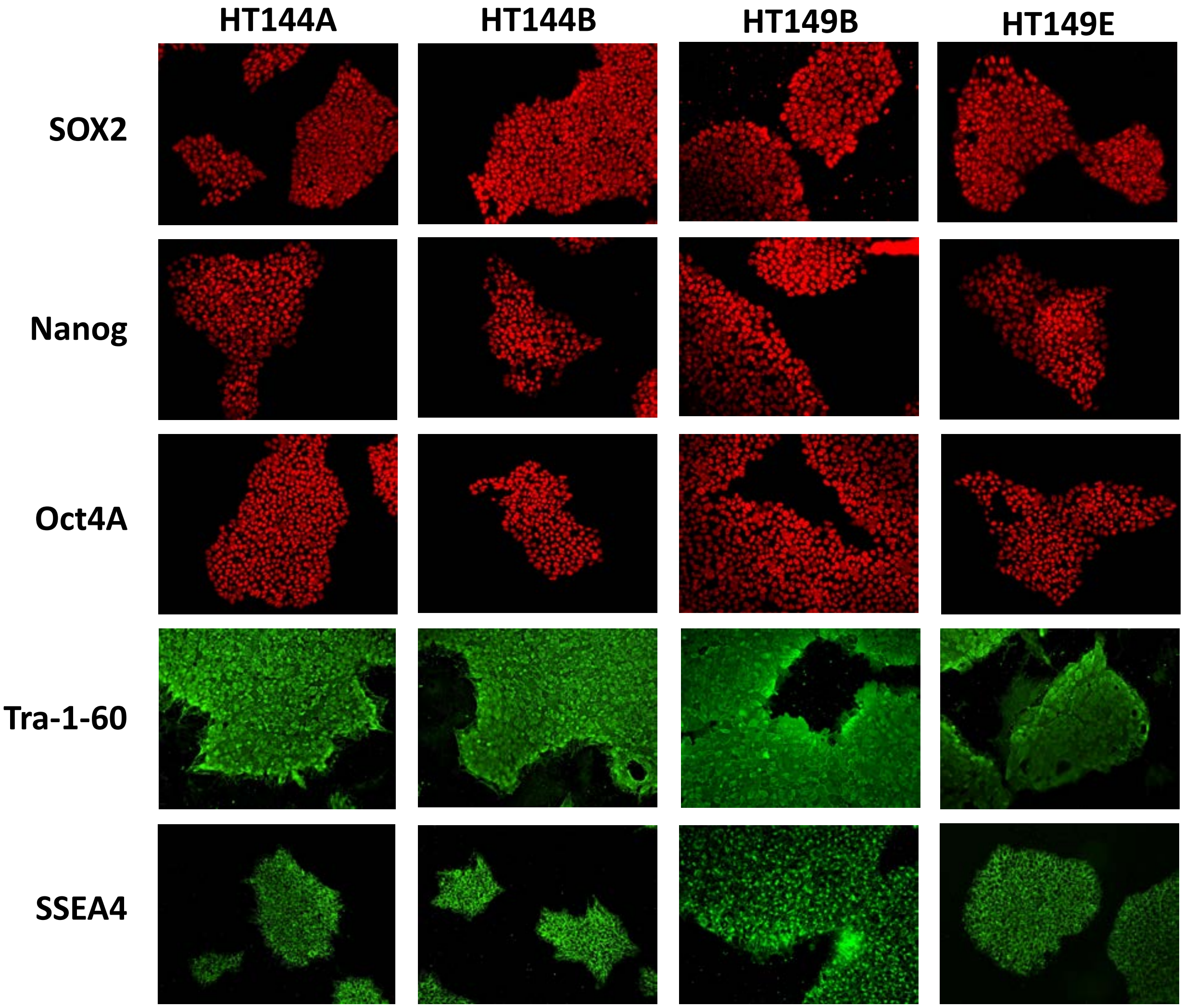

Figure S1. Immunocytochemical characterization of WD iPSCs.

WD iPSCs express typical pluripotency protein markers SOX2, Nanog, OCT4, Tra-1-60, and SSEA4.

Cell line	Fibroblast GM06144	iPSC HT144A	iPSC HT144B	NSC HT144A	NSC HT144B	Fibroblast GM11851	iPSC HT149B	iPSC HT149E	NSC HT149B	NSC HT149E
FGA	22,23	22,23	22,23	22,23	22,23	21,21	21,21	21,21	21,21	21,21
TPOX	10,10	10,10	10,10	10,10	10,10	8,8	8,8	8,8	8,8	8,8
D8S1179	12,13	12,13	12,13	12,13	12,13	13,13	13,13	13,13	13,13	13,13
vWA	19,19	19,19	19,19	19,19	19,19	17,17	17,17	17,17	17,17	17,17
Amelogenin	X,Y	X,Y	X,Y	X,Y	X,Y	X,X	X,X	X,X	X,X	X,X
Penta_D	14,14	14,14	14,14	14,14	14,14	9,11	9,11	9,11	9,11	9,11
CSF1PO	13,13	13,13	13,13	13,13	13,13	10,12	10,12	10,12	10,12	10,12
D16S539	11,11	11,11	11,11	11,11	11,11	11,13	11,13	11,13	11,13	11,13
D7S820	9,10	9,10	9,10	9,10	9,10	10,12	10,12	10,12	10,12	10,12
D13S317	11,14	11,14	11,14	11,14	11,14	9,12	9,12	9,12	9,12	9,12
D5S818	12,13	12,13	12,13	12,13	12,13	11,12	11,12	11,12	11,12	11,12
Penta_E	15,16	15,16	15,16	15,16	15,16	11,13	11,13	11,13	11,13	11,13
D18S51	15,16	15,16	15,16	15,16	15,16	14,15	14,15	14,15	14,15	14,15
D21S11	30,32.2	30,32.2	30,32.2	30,32.2	20,32.2	28,29	28,29	28,29	28,29	28,29
TH01	7,9.3**	7,9.3	7,9.3	7,9.3	7,9.3	6,7	6,7	6,7	6,7	6,7
D3S1358	17,17	17,17	17,17	17,17	17,17	14,16	14,16	14,16	14,16	14,16

Figure S2. STR DNA analysis of WD fibroblasts, iPSCs, and NSCs.

STR DNA analysis confirmed that the iPSC and NSC lines generated in the lab could be traced back to the original fibroblast lines from which they were derived.

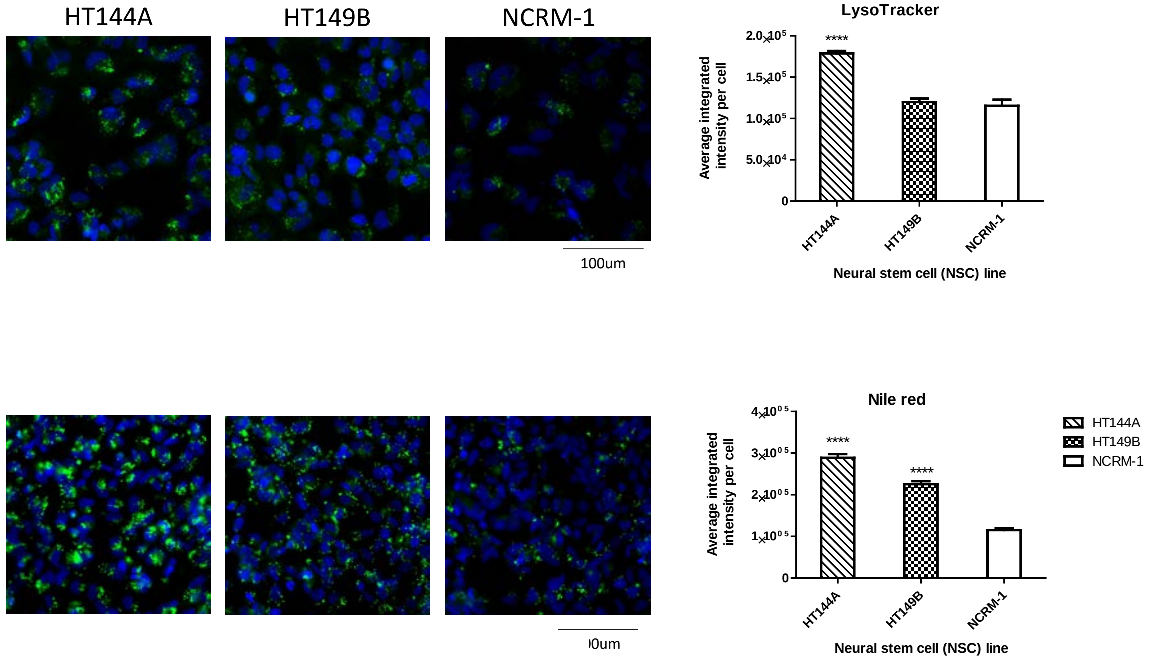

Figure S3. LysoTracker staining and Nile red staining of LDL loaded NSCs

(A) LysoTracker (green) and nuclear (blue) staining images with quantitation of LDL treated NSCs. (B) Nile red (green) and nuclear (blue) staining images with quantitation of LDL treated NSCs. All data are displayed with mean ± SD. Statistical significance calculated as each WD cell line against NCRM-1.

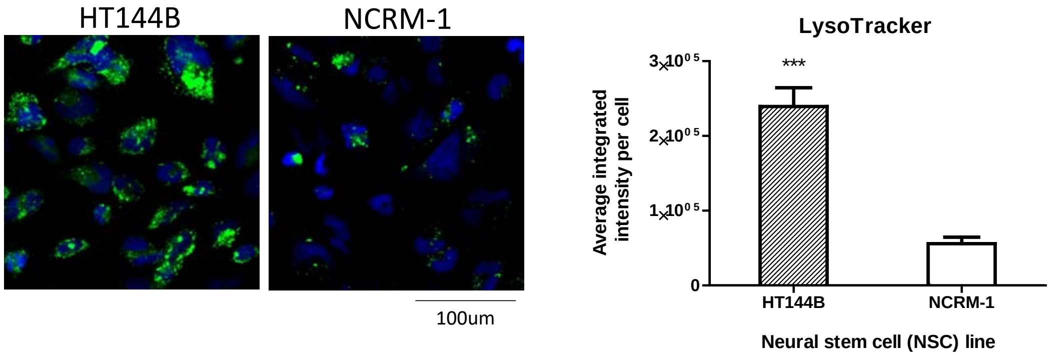

Figure S4. HT144B NSCs show increased LysoTracker staining

LysoTracker (green) and nuclear (blue) staining images with quantitation of WD and control NSCs. All data are displayed with mean ± SD. Statistical significance calculated as WD cell line against NCRM-1.

A. Delta-Tocopherol (DT)

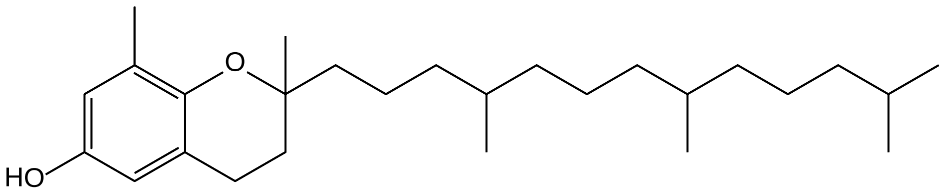

B. Hydroxypropyl beta-Cyclodextrin (HPBCD)

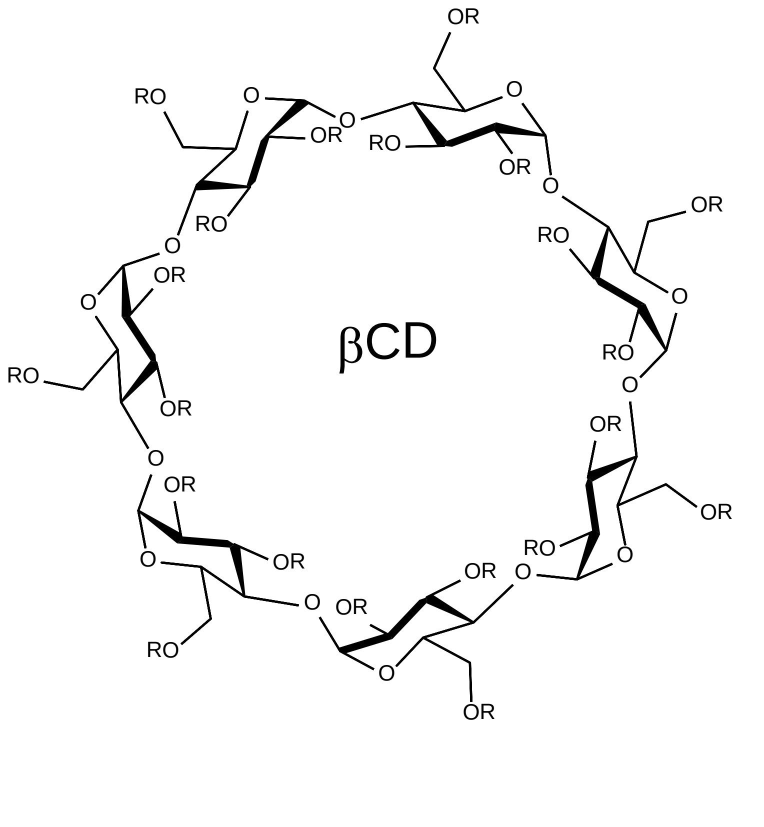

Figure S5. Chemical structures

(A) Chemical structure of DT. (B) Chemical structure of HPBCD.

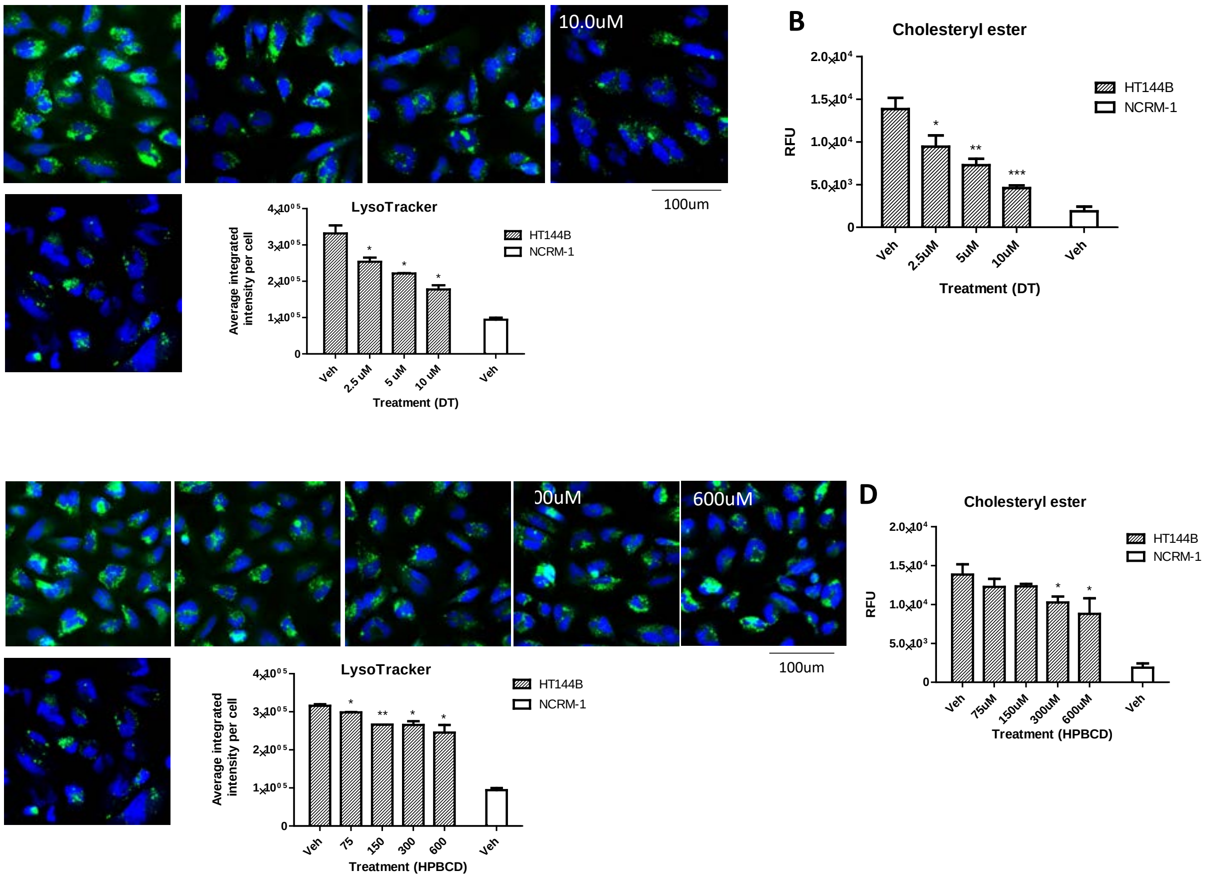

Figure S6. DT and HPBCD treatment reduces lysosomal staining in HT144B NSCs.

(A) LysoTracker (green) and nuclear (blue) staining images with quantitation of DT treated NSCs. (B) Amplex red assay for cholesteryl ester content of NSCs treated with DT. (C) LysoTracker (green) and nuclear (blue) staining images with quantitation of HPBCD treated NSCs. (D) Amplex red assay for cholesteryl ester content of WD NSCs treated with HPBCD. Data are displayed with mean ± SD. Statistical significance calculated as each concentration of compound compared against vehicle in the same WD cell line.

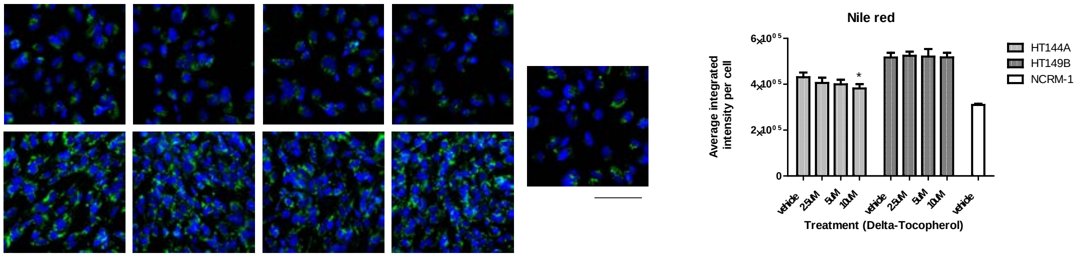

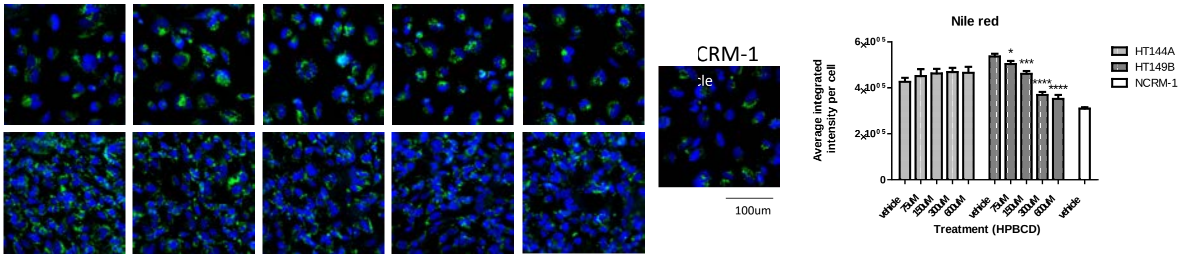

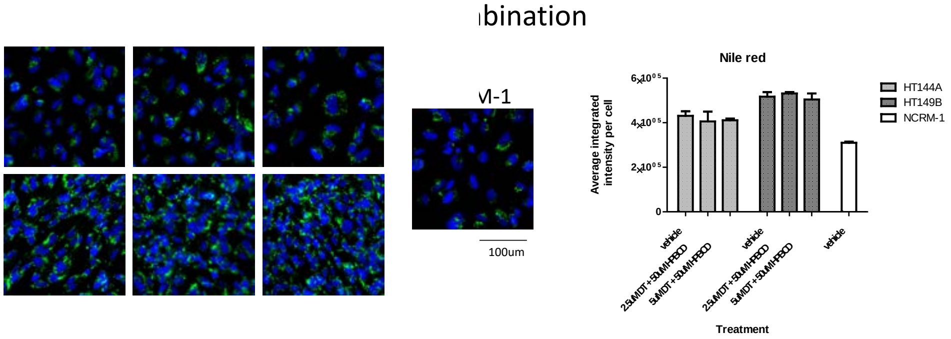

Figure S7: DT and HPBCD do not significantly reduce neutral lipid accumulation in WD NSCs. (A) Nile red (green) and nuclear (blue) staining images with quantitation of DT treated NSCs. (B) Nile red (green) and nuclear (blue) staining images with quantitation of HPBCD treated NSCs. (C) Nile red (green) and nuclear (blue) staining images with quantitation of combination treated NSCs. Data are displayed with mean ± SD. Statistical significance calculated as each treatment compared against vehicle treatment in the same WD cell line.

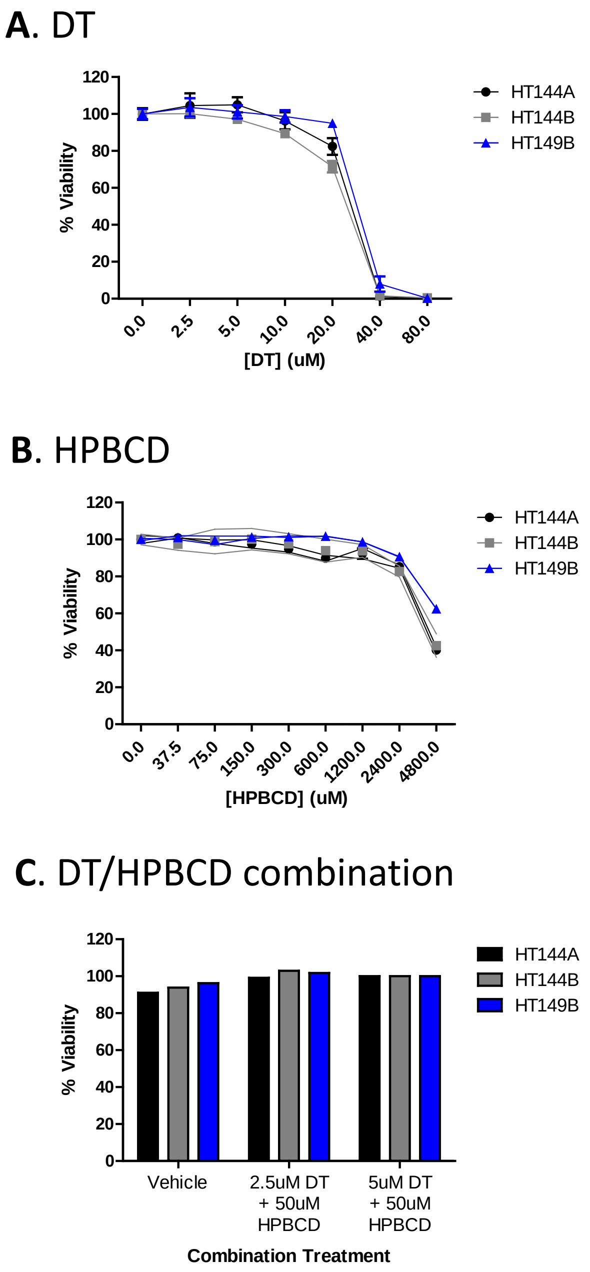

Figure S8: High concentrations of DT and HPBCD affect cell viability.

(A) ATP content cell viability assay of NSCs treated with DT. (B) ATP content cell viability assay of NSCs treated with HPBCD. (C) ATP content cell viability assay of NSCs treated with combination DT and HPBCD.

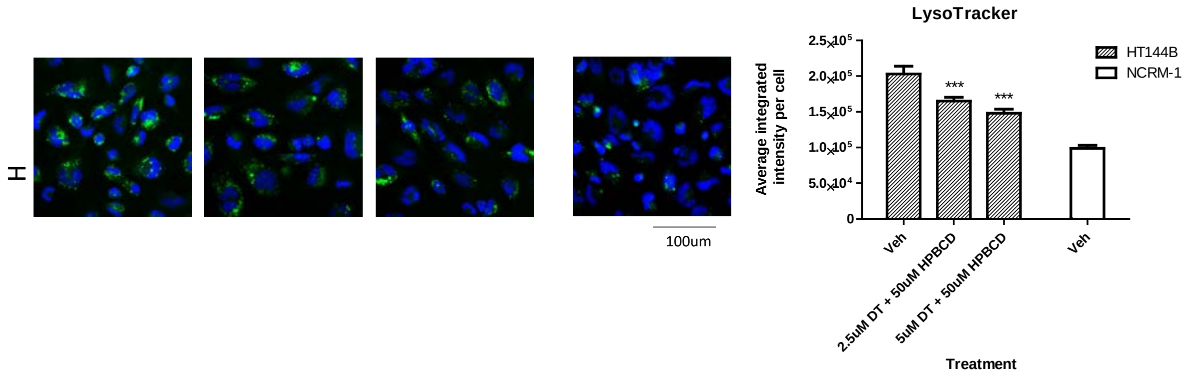

B. Cholesterol ester assay for HPBCD and DT combination

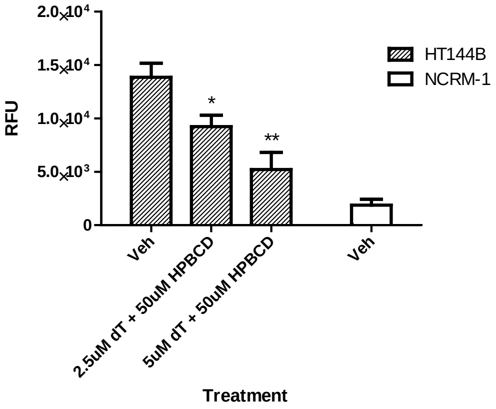

Figure S9: DT and HPBCD combination treatment have an additive effect on reducing lysosomal staining in HT144B NSCs.

(A) LysoTracker (green) and nuclear (blue) staining images with quantitation of DT and HPBCD combination treatment. (B) Amplex red assay for cholesteryl ester content of WD NSCs treated with DT and HPBCD combination. Data are displayed with mean ± SD. Statistical significance calculated as each combination treatment compared against vehicle treatment in the same WD cell line.
